# Season’s Cheatings: Beware of Holiday Scams

**DOI:** 10.1093/geroni/igaa057.1441

**Published:** 2020-12-16

**Authors:** Lona Choi-Allum, Alicia Williams

**Affiliations:** AARP, Washington, District of Columbia, United States

## Abstract

‘Tis the season…to be on the lookout for possible scams and fraud. It’s during this time of year that individuals are more focused on the spirit of the holidays and less focused on what may be happening with their pocketbook. AARP conducted a survey of 2,842 U.S. adults ages 18 and older to understand people’s awareness of and experience with a variety of scams that are common around the holidays. The study explored experiences with purchasing gift cards, shipping/receiving packages, and charitable giving. In addition, the survey tested the knowledge of adults about several specific scams with a ‘quiz’ of five true or false statements. Results showed that one in six (17%) U.S. adults failed the quiz. When making gift card purchases, one in five U.S. adults have given and/or received a gift card that had no funds on it. And only about half of U.S. adults conduct research before making a monetary donation to charitable causes or organizations. Of those who do check out a charity first, over half (54%) did not make a donation based on what they found on charity rating sites. Also, U.S. adults say that packages are left outside of their home without requiring a signature. Half (50%) of U.S. adults say they never require a signature when shipping packages to home addresses while about one in seven (15%) always require a signature. More education is needed to raise awareness of scams that are common during the holidays.

